# Enhancing Nitrate Removal from Freshwater Pond by Regulating Carbon/Nitrogen Ratio

**DOI:** 10.3389/fmicb.2017.01712

**Published:** 2017-09-08

**Authors:** Rong Chen, Min Deng, Xugang He, Jie Hou

**Affiliations:** ^1^College of Fisheries, Huazhong Agricultural University Wuhan, China; ^2^School of Environmental Studies, China University of Geosciences Wuhan, China; ^3^Freshwater Aquaculture Collaborative Innovation Center of Hubei Province Wuhan, China; ^4^Hubei Provincial Engineering Laboratory for Pond Aquaculture Wuhan, China

**Keywords:** C/N ratio, denitrification, functional gene, community structure, freshwater ponds

## Abstract

Nitrogen accumulation is a serious environmental problem in freshwater ponds, which can lead to massive death of fish and shrimps as well as the eutrophication. The removal of nitrate by regulating the carbon to nitrogen (C/N) ratio and the underlying mechanisms were investigated. The nitrate removal system comprised 530-mL medium containing 5 mg/L ${NO}_{3}^{-}$-N and 0–66.6 mg/L COD (i.e., C/N ratio of 0–13.3) and 20 g ponds sediments. When the C/N ratio was higher than 8, the nitrate removal efficiency nearly reached 100% during the incubation period and the accumulation of nitrite was negligible. When the C/N ratio was below 8, the nitrate removal efficiency was lower and significant nitrite accumulation occurred. The nitrate removal rate increased with the C/N ratio increased, which was ascribed to the increase in the absolute abundance of denitrifiers (*nirS*, *nirK*, and *nosZ*). Although both *nirS*-type and *nirK*-type denitrifiers were found in the sediments of freshwater pond, *nirS*-type denitrifiers were predominant. *Dechloromonas* was the major *nirS*-type denitrifier for nitrate removal in *nirS*-type with the C/N ratios above 5.33, while the majority of the *nirK*-type denitrifiers were unclassified. Thus, this study implied that the appropriate C/N ratio played an important role on the removal of excess nitrate from freshwater ponds.

## Introduction

Freshwater ponds provide abundant food resources to humans, especially in China, supplying nearly 20 million tons of fishery production every year. Recently, high density and intensive farming models with superfluous feeding and fertilization have been used to achieve higher economic efficiency, resulting in nitrogen accumulation owing to excessive residual feed and excrement (Avnimelech and Ritvo, 2003). Although nitrogen is an important nutrient for aquatic organisms such as fish, excess nitrogen in freshwater ponds leads to the massive death of fish and shrimps, and the eutrophication (Camargo and Alonso, 2006). For instance, nitrite (${NO}_{2}^{-}$), a reductive product of nitrate (${NO}_{3}^{-}$), is toxic to aquatic organisms due to the damage to hemoglobin (Camargo and Alonso, 2006). In addition, contaminated pond water also affects the water quality of the surrounding larger water bodies such as lakes (Cao et al., 2007). In ponds, nitrate, organic nitrogen, and ammonium nitrogen (NH_4_^+^-N) are the predominant nitrogen species. While nitrification couples the conversion of NH_4_^+^-N to nitrate (Wu et al., 2009), denitrification reduces nitrate to nitrogen (Kraft et al., 2014), further releasing nitrogen into the atmosphere. Aquatic animals require feed with a high protein concentration (Hari et al., 2006) of up to 50% (Wei et al., 2016). About 75% of nitrogen in feed ends up in water through ammonification of uneaten feed and excretion (Crab et al., 2012). The high concentration of nitrogen in water limits the transformation capacity of denitrification in natural ponds because denitrifiers are mainly heterotrophic microorganisms requiring high carbon content for their growth (Crab et al., 2012). The excessive accumulation of nitrogen will spoil the living environment of the aquatic animals (Hari et al., 2006). Hence, it is a major environmental concern to develop cost-effective processes for controlling nitrogen in contaminated freshwater ponds.

Previous studies have shown that ion exchange, reverse osmosis, electrochemical processes, and biological treatment can effectively remove nitrate from wastewater (Shrimali and Singh, 2001; Koparal and Ogutveren, 2002; Mohan et al., 2016). However, the regeneration of anion exchange resin requires a high amount of regenerant for ion exchange, the discharge of brine and high concentrations of nitrate during reverse osmosis may lead to secondary pollution, and the cost of power for electrochemical processes is very high. Consequently, biological treatment has attracted increasing attention in recent years owing to its higher efficiency and lower cost (Shrimali and Singh, 2001; Mohan et al., 2016). Constructed wetlands and sequencing batch reactors (SBRs) are commonly used for nitrate removal from wastewater (Wu et al., 2009; Mohan et al., 2016) through denitrification to achieve the conversion of nitrate to nitrogen. The redox environment often occurs in anoxic conditions at the bottom of freshwater ponds, which is beneficial for the removal of nitrate by enhancing the denitrification efficiency. Although wastewater treatment plants (WWTPs) have exhibited ideal nitrate removal efficiency through denitrification and their underlying mechanisms have been extensively explored (Zhao et al., 2013; Mannina et al., 2016), the microbial communities in activated sludge applied to WWTPs are different from those in freshwater ponds. As a result, the denitrification mechanism in freshwater ponds may differ from that in WWTPs. In addition, WWTPs just require the absence of nitrite in the effluent water, and nitrite accumulation during the intermediate process can be ignored. As nitrite is toxic to aquatic organisms (Camargo and Alonso, 2006), its accumulation cannot occur during the entire process of denitrification in freshwater ponds.

Denitrification, an important nitrogen removal mechanism that can ameliorate the effects of nitrogen pollution via conversion of nitrate to nitrogenous gas (Kraft et al., 2014; Ward and Jensen, 2014; Morrissey and Franklin, 2015), is the major pathway for the removal of nitrogen from water bodies (Altabet et al., 1995; Hargreaves, 1998; Laverman et al., 2010). Previous studies have shown that several factors, including temperature, pH, dissolved oxygen (DO) level, organic carbon species, and ratio of organic carbon to nitrogen (C/N), can affect the denitrification efficiency (Strong et al., 2011; Kraft et al., 2014). In freshwater ponds, the temperature varies along with ambient environment and the pH was maintained in the range of 6–8, and these factors are difficult to adjust by manual operation. In contrast, the type of organic matter and C/N ratio can be easily regulated by daily feed. The C/N ratio has been identified as a key environmental factor that determines the products of nitrate reduction (Kraft et al., 2014). Therefore, it is rational to hypothesize that we can obtain higher denitrification efficiency by regulating the C/N ratio through the addition of extra organic carbon.

In the present study, the effect and extent of the impact of different C/N ratios on the nitrate removal efficiency in ponds sediments were investigated. For this purpose, denitrifiers from the sediments of freshwater ponds were cultured in nutrient medium with different C/N ratios for 30 days, and the variation in nitrate content during the incubation period was explored. Three functional genes, *nirS*, *nirK*, and *nosZ*, were employed to measure the abundance and community structure (without *nosZ*) of the denitrifiers by using real-time quantitative polymerase chain reaction (qPCR) technology and high-throughput sequencing technology, respectively, at different time points during the incubation period. The results obtained could help in understanding the role of C/N ratio in nitrate removal and the variations in the abundance and community structure of denitrifiers, facilitating the development of efficient technology for nitrogen removal from freshwater ponds.

## Materials and Methods

### Chemicals and Sediments

KCl, NaH_2_PO_4_, Na_2_HPO_4_, CH_3_COONa (NaAc), and KNO_3_ were above analytical grade and purchased from Sinopharm Chemical Reagent Co., Ltd, China. The sediment samples were collected from a fishpond located in Gong’an, Hubei province, China (112°15′44.63″E, 29°55′14.62″N). About 5 kg of sediment was collected at 0.2 m below the surface of the pond using a UWITEC Sediment corer 60 (Mondsee, Austria) on April 12, 2014, and transported to the laboratory at 4°C. Then, the sediment was washed with 1 mol/L KCl solution to decrease the ammonia concentration to below 0.5 mg/L, washed with 1 mol/L phosphate-buffered saline (PBS, pH = 7.0) to remove potassium ions, and centrifuged at 6000 *g* for 10 min.

### Batch Experiments

All the experiments were conducted at 25 ± 2°C in 550-mL glass bottle reactors wrapped by aluminum foils to avoid the light. Every glass reactor was sealed by using a suitable bottle cap with a seal ring to assure good sealing. Prior to the experiment, 20 g of pretreated sediment samples were suspended in 530 mL of medium containing 20 mM PBS (pH 7.5) and 0.2 mg/L trace elements, including Cu, Fe, Mn, Ca, Mg, and Co (Bi et al., 2015). The suspension was purged with nitrogen gas (99.999%) for 30 min to remove oxygen. To start the experiment, medium containing 5 mg N/(L⋅d) with different concentrations of organic carbon [0, 13.35, 26.65, 40, and 66.65 mg COD/(L⋅d) supplied by NaAc] were added to the suspension. The hydraulic retention time (HRT) was 1 day (24 h) during the entire operation period (30 days). After addition of the influent, the reactors were purged with nitrogen gas (99.999%) for 30 min. Each experiment was conducted in triplicate, and the control experiments were performed with sterile water and sediment samples.

To obtain an insight into the process of nitrate removal, the kinetics of nitrate reduction was determined. A total of 300 g of pretreated sediment were respectively added to two reactors (1100 mL) containing 950 mL of the medium [5 mg N/(L⋅d) and 40 mg COD/(L⋅d)]. On day 15, the sediment samples were collected from the two reactors and centrifuged at 6000 *g* for 10 min. Subsequently, 20 g of the centrifuged sediment were added to the reactor containing 530 mL of the medium (5 mg/L ${NO}_{3}^{-}$-N with different concentrations of organic carbon 0, 13.35, 26.65, 40, and 66.65 mg/L COD, supplied by NaAc). The medium in each reactor was bubbled with nitrogen gas (99.999%) for 30 min after sampling. On day 30, the sediment samples were collected from the two reactors and conducted with the same treatment as on day 15. Each experiment was performed in triplicate, and the control comprised sterile water and sediment samples. At predetermined time points (days 0, 15, and 30), about 8 mL of the medium were filtered through a 0.45-μm membrane, centrifuged at 6000 *g* for 10 min, and about 2 g of the sediment were collected and stored at -80°C for DNA extraction.

### DNA Extraction and qPCR

Soil DNA kits D5625-01 (Omega, United States) were used to extract and purify the total genomic DNA from the samples. The extracted genomic DNA was detected by 1% agarose gel electrophoresis and stored at -20°C until further use. The target fragments of *nirK*, *nirS*, and *nosZ* were subjected to PCR, and all the primers used were synthesized by TSINGKE Biotechnology Co. (Wuhan, China) and diluted to a concentration of 10 mmol/L (Supplementary Table S1). The PCR products were cloned into the pMD18-T Easy Vector (Takara, Dalian, China). The plasmids containing specific functional genes (i.e., *nirK*, *nirS*, and *nosZ*) were obtained from TSINGKE Biotechnology Co. (Beijing, China). The standard samples were diluted to yield a series of 10-fold concentrations and subsequently used for constructing qPCR standard curves. The *R*^2^ value for each standard curve exceeded 0.99, indicating good linear relationships over the concentration ranges used in this study. The amplification efficiency for each standard curve was between 98 and 102%.

qPCR was performed on a Qiagen Q thermocycler (Qiagen, Germany) with 20-μL reaction mixture containing 10 μL of SYBR Green II PCR master mix (Takara), 1 μL of template DNA (sample DNA or plasmid DNA for standard curves), forward and reverse primers (Supplementary Table S2), and sterile water (Millipore, United States). The reaction was performed using a three-step thermal cycling procedure, and the protocol and parameters for each target gene are presented in Supplementary Table S2. Each qPCR comprised 40 cycles, followed by a melting curve analysis. All the measurements were performed in triplicate. Sterile water was used as a negative control, and the qPCR data were normalized to copies/g dry sediment.

### Illumina MiSeq Sequencing and Data Analysis

PCR and sequencing were conducted as described in a previous study (Caporaso et al., 2012) with primers nirScd3aF–nirSR3cd and nirKFlaCu–nirKR3Cu for amplifying *nirS* and *nirK*, respectively. Sequencing was performed using MiSeq Benchtop Sequencer (Illumina, United States) by Shanghai Majorbio Bio-pharm Technology Co., Ltd. (Shanghai, China), and the sequencing data were analyzed using Mothur software (Schloss et al., 2009).

### Analysis

Every 5 days, the DO level and pH of the influent and effluent were measured by using DO 2000 LDOTM (Thermo Eberline Trading GmbH, Wermelskirchen, Germany) and HI-9025 pH meter (Hanna, Padova, Italy), respectively. The DO level and pH of the influent and effluent ranged between 0.32 and 0.48 mg/L and between 7.18 and 7.46, respectively. The NH_4_^+^-N concentration in the influent and effluent ranged from 0.28 to 0.47 mg/L.

The concentrations of NH_4_^+^-N, nitrite-nitrogen (${NO}_{2}^{-}$-N), and nitrate-nitrogen (${NO}_{3}^{-}$-N) were determined by using NanoDrop 2000 UV-Vis spectrophotometer (Thermo Fisher Scientific, New York, United States) according to standard analytical procedures (Water Environment Federation, 2005). Specifically, the concentrations of NH_4_^+^-N, ${NO}_{2}^{-}$-N, and ${NO}_{3}^{-}$-N were measured spectrophotometry using Nessler’s reagent, *N*-(1-naphthyl)ethylenediamine dihydrochloride, and UV spectrophotometry using hydrochloric acid, respectively. The concentration of nitrogen species in the influent and effluent and HRT (24 h) were used to calculate the ${NO}_{2}^{-}$-N removal efficiency and accumulation rate. Correlation coefficients were calculated to evaluate the associations between C/N ratio and nitrate removal efficiency, accumulation of ${NO}_{2}^{-}$-N, and nitrogen transformation genes. The C/N ratio was referred to the adding COD to the nitrate.

The denitrification kinetics was determined with the first-order model as follows: *S* = *S*_0_⋅e^-^*^k^*^.^*^t^* (Simkins and Alexander, 1984; Tiemeyer et al., 2007), where *k* is the denitrification rate constant [mg/(L⋅h)], *t* is the reaction time (h), and *S*_0_ and *S* (mg/L) are the concentrations of ${NO}_{3}^{-}$-N at reaction time 0 and *t*, respectively.

### Nucleotide Sequence Accession Numbers

The nucleotide sequences obtained in this study were deposited in the GenBank database under accession nos. KR232567–KR232570 for *nirS* and KP262401 for *nirK*.

## Results and Discussion

### Effect of C/N Ratio on Nitrate Removal

**FIGURE 1:**
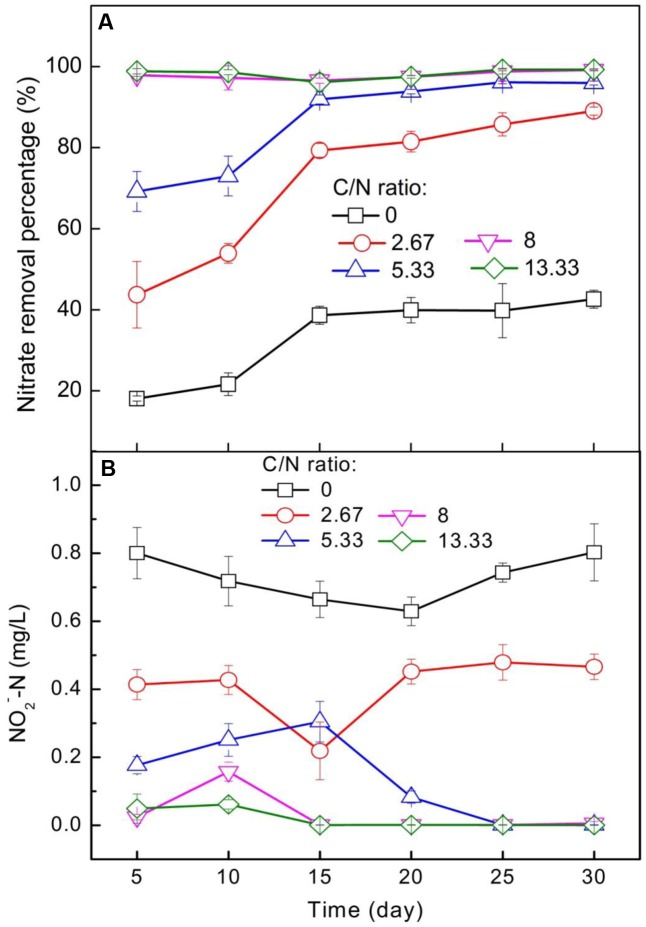
Effects of C/N ratio on **(A)** nitrate removal efficiency and **(B)** accumulation of nitrite.

In the absence of extra organic carbon (C/N ratio = 0), the removal efficiency of nitrate increased from 18.1 to 42.6% in 30 days (**Figure 1A**). The decrease in nitrate during the incubation period may be due to the denitrification using pristine organic carbon in the sediments. Interestingly, a remarkable increase in nitrate removal efficiency was observed with the addition of extra organic carbon (**Figure 1A**). When the C/N ratio was less than 8, the nitrate removal efficiency increased from 43.7 to 69.2% on day 5 and reached 89 and 96% on day 30 with the increase in C/N ratio from 2.67 to 5.33. However, nitrate could not be efficiently removed when the C/N ratio was 0–2.67, which was reasonably lower than the theoretically as well as experimentally determined value of 2.86 and 3.5–4.5 for complete denitrification, respectively (Henze et al., 1994). When the C/N ratio was higher than 8, the nitrate removal efficiency nearly reached 100% during the entire incubation period, which may be owing to the presence of adequate organic carbon for denitrification (Cervantes et al., 2001). Thus, the increase in nitrate removal efficiency with the increase in C/N ratio confirmed the hypothesis that nitrate removal is mediated by C/N ratio. Furthermore, correlation analysis showed a positive correlation between C/N ratio and average nitrate removal efficiency (*r* = 0.919, *P* < 0.01) on day 15 (Supplementary Table S3), and similar results were also found a tother time points with correlation coefficients above 0.7 and a significance level below 0.01 (Supplementary Table S3). These results further support the association between nitrate removal and C/N ratio.

As ${NO}_{2}^{-}$ is the initial product of denitrification and is toxic to aquatic organisms, the variation of ${NO}_{2}^{-}$ was measured during the incubation period (**Figure 1B**). In the absence of extra organic carbon (C/N ratio = 0), significant accumulation of ${NO}_{2}^{-}$-N was observed within 30 days. The concentration of ${NO}_{2}^{-}$-N was nearly stabilized at 0.8 mg/L, amounting to 16% of the initial ${NO}_{3}^{-}$-N concentration (5 mg/L). The nitrate removal efficiency ranged from 18.1 to 42.6% at the C/N ratio of 0 (**Figure 1A**), suggesting that some nitrate was reduced to nitrite (**Figure 1B**). However, nitrite is toxic to denitrifiers (Tiemeyer et al., 2007; Wang Y. et al., 2014), and thus may further inhibit the denitrification step (${NO}_{2}^{-}$ → NO or N_2_O) by decreasing the activity of related enzymes. With the increase in C/N ratio from 2.67 to 13.33, the concentration of ${NO}_{2}^{-}$-N decreased from 0.41 to 0.05 on day 5 and from 0.47 to undetectable levels (the detection limit for ${NO}_{2}^{-}$-N was 0.003 mg/L) on day 30. The notable decrease in ${NO}_{2}^{-}$-N concentration at higher C/N ratios suggested that the addition of extra organic carbon is necessary for reducing the risk of nitrite poisoning in freshwater ponds. Correlation analysis showed a negative correlation between ${NO}_{2}^{-}$-N and C/N ratio (*r* > 0.8, *P* < 0.01) during the incubation period (Supplementary Table S4). Previous studies had demonstrated that the formation and accumulation of nitrite were controlled by the C/N ratio (Hari et al., 2006; Zhi and Ji, 2014). For example, for the low-carbon-concentration treatment (37.7% carbohydrates), the concentrations of nitrite and nitrate were 6.5- and 6.9-fold higher than those in the high-carbon-concentration treatment (about 47.6% carbohydrates) (Wei et al., 2016). The decrease in the concentrations of nitrate and nitrate in the presence of high concentrations of carbon may be attributed to denitrification. Hari et al. (2006) and Avnimelech (1999) observed a similar effect of carbon concentration on the concentrations of nitrite and nitrate. The decrease in the concentrations of nitrite and nitrate is beneficial to aquatic animals because toxic nitrite can lead to low survival rate or decreased growth (Wei et al., 2016).

To determine the optimal C/N ratio, the nitrate removal efficiency and cost should be comprehensively considered. A high C/N ratio could cause energy waste and lead to ammonification (Kraft et al., 2014), whereas a low C/N ratio could result in deficiency in nitrate removal and accumulation of nitrite. In the present study, the complete removal of nitrate and negligible accumulation of nitrite were achieved at a C/N ratio of 8–13.33, revealing that a C/N ratio above 8 is required to avoid poisoning of aquatic organisms by achieving complete denitrification (without ${NO}_{2}^{-}$-N accumulation). The results of this study are similar to those reported in previous studies, which achieved optimal nitrogen removal (in SBR system) at a C/N ratio of 11.1 and 11.2 (Münch et al., 1996; Chiu et al., 2007).

### Kinetics of Nitrate Removal at Different C/N Ratios

**FIGURE 2:**
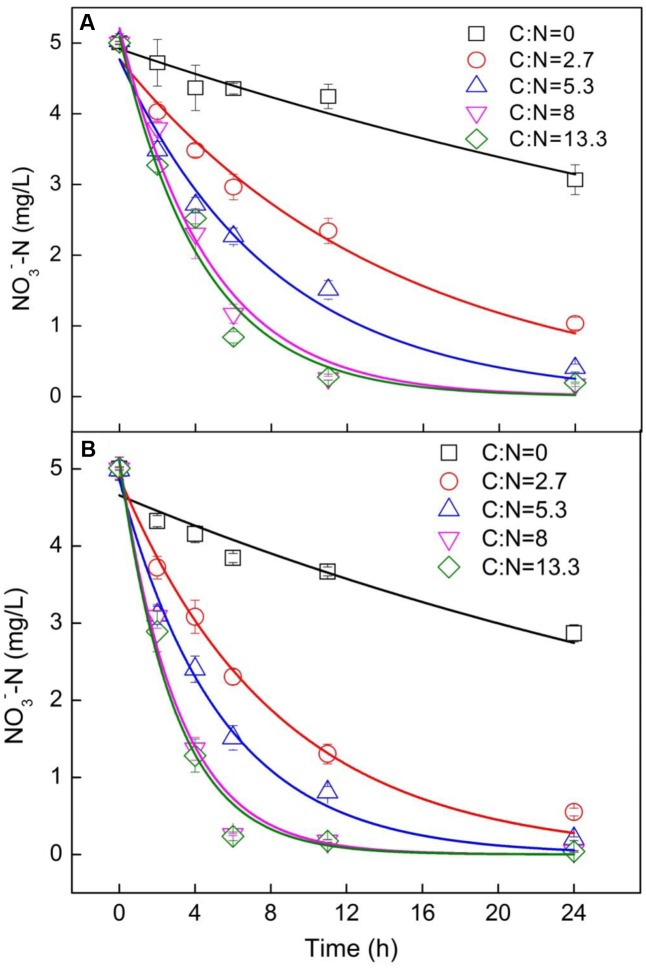
Kinetic models of nitrate removal with time on **(A)** day 15 and **(B)** day 30. A first-order kinetic model was used to fit the decrease in nitrate with time.

To get further insight into the influence of C/N ratio on nitrate removal, the kinetics of nitrate removal on days 15 and 30 was explored (**Figure 2**). On day 15, the concentration of ${NO}_{3}^{-}$-N decreased from 5 to 0.2 mg/L in 24 h with the increase in C/N ratio from 0 to 13 (**Figure 2A**). The decrease in ${NO}_{3}^{-}$-N followed first-order kinetics, with rate constants of 0.02, 0.07, 0.12, 0.21, and 0.23 mg/(L⋅h) for systems with C/N ratios of 0, 2.67, 5.33, 8, and 13.3, respectively (**Table 1**). The positive correlation of denitrification rate constants with C/N ratio (*r* = 0.96, *P* = 0.01, Supplementary Figure S1a; *r* = 0.94, *P* = 0.02, Supplementary Figure S1b) further confirmed that denitrification is dependent on C/N ratio. On day 30, the rate constants were 0.022, 0.12, 0.19, 0.32, and 0.34 mg/(L⋅h) for systems with C/N ratios of 0, 2.67, 5.33, 8, and 13.3, respectively (**Table 1**), which were higher than those noted on day 15. The increase in the denitrification rate with the incubation was presumably due to the increase in the population of denitrifiers with the incubation time. Previous studies have shown that the increase in C/N ratio can stimulate bacterial growth (Inwood et al., 2007), subsequently improving the denitrification efficiency per cell. Accordingly, in the present study, the variations in the abundance of functional genes, diversity, and community of denitrifiers were examined.

**Table 1:** Kinetic parameters of the different nitrate concentration.

Group	15th day		30th day	
		
	*k* [mg/(L⋅h)]	*R*^2^	*k* [mg/(L⋅h)]	*R*^2^
C:N = 0	0.019 ± 0.0024	0.94	0.022 ± 0.0037	0.89
C:N = 2.67	0.070 ± 0.0062	0.98	0.12 ± 0.0084	0.99
C:N = 5.33	0.12 ± 0.014	0.97	0.19 ± 0.014	0.99
C:N = 8	0.21 ± 0.024	0.98	0.32 ± 0.040	0.97
C:N = 13.33	0.23 ± 0.030	0.97	0.34 ± 0.036	0.98


### Effect of C/N Ratio on the Absolute Abundance of Functional Genes

**FIGURE 3:**
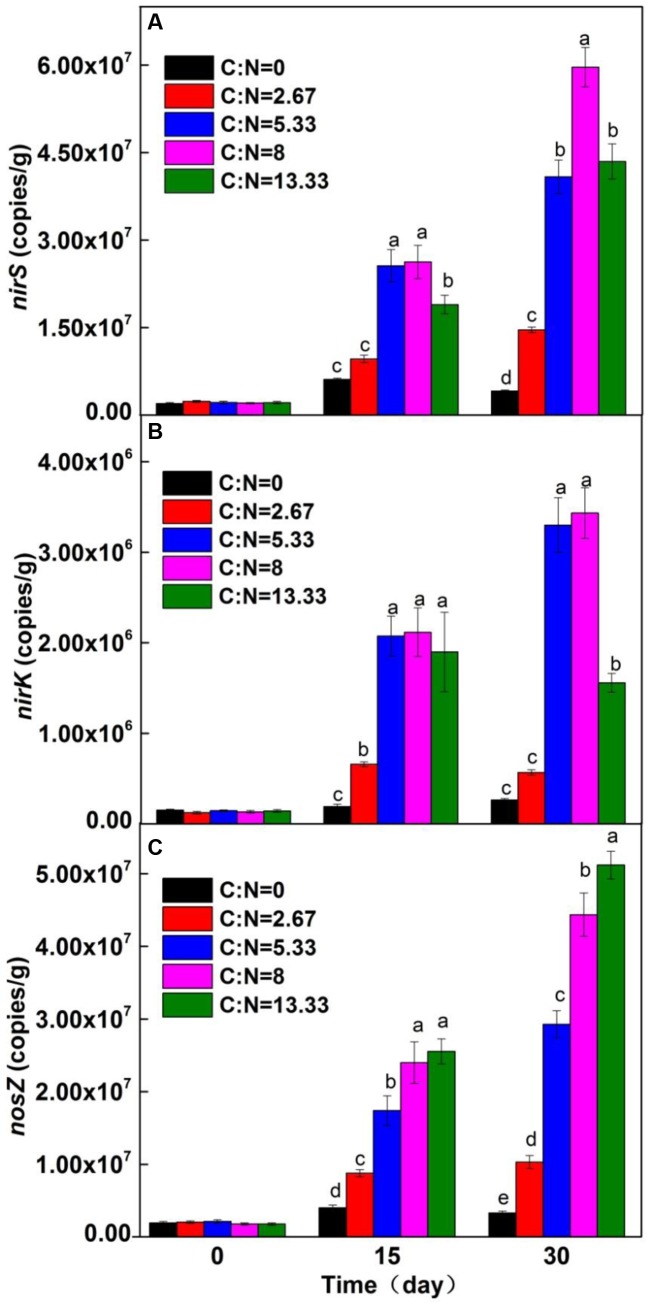
Absolute abundance of functional genes: **(A)** *nirS*, **(B)** *nirK*, and **(C)** *nosZ*. Different letters (a–e) represent samples with a significant difference at the level of *P* = 0.05 (one-way ANOVA).

Nitrite reduction, the symbolic and key step of denitrification, is catalyzed by nitrite reductases, including copper-containing and multiheme enzymes encoded by the genes *nirK* and *nirS*, respectively (Stouthamer, 1992; Fan et al., 2015). As nitrous oxide reduction is the last step of denitrification, the related functional gene *nosZ* is usually utilized to estimate complete denitrification (Kandeler et al., 2006; Bowles et al., 2012). In the present study, to illustrate the influence of C/N ratio on growth of denitrifiers, the abundances of *nirK*, *nirS*, and *nosZ* were measured during the incubation period (**Figure 3**). In the absence of extra organic carbon (C/N ratio = 0), the negligible increase in the abundances of *nirK*, *nirS*, and *nosZ* implied slow growth of denitrifiers. In contrast, with the addition of extra organic carbon, the abundances of these functional genes observably increased with the incubation period. The abundances of *nirS*, *nirK*, and *nosZ* increased from 1.98 × 10^6^ to 5.96 × 10^7^ copies/g (**Figure 3A**), from 1.23 × 10^5^ to 3.43 × 10^6^ copies/g (**Figure 3B**), and from 3.30 × 10^6^ to 5.12 × 10^7^ copies/g (**Figure 3C**), respectively, indicating the growth of microbial population. The positive correlation between the abundances of *nirS*, *nirK*, and *nosZ* and C/N ratio increased on days 15 and 30 (**Table 2**), further affirming that the C/N ratio plays a critical role in the growth of denitrifiers.

**Table 2:** Correlation between gene abundance and C/N ratio.

Gene	15th day		30th day	
		
	Correlation coefficient	Significance	Correlation coefficient	Significance
*nirS*	0.61	0.02	0.77	0.00
*nirK*	0.76	0.00	0.46	0.08
*nosZ*	0.93	0.00	0.96	0.00


At C/N ratios below 8, the absolute abundances of *nirS*, *nirK*, and *nosZ* significantly increased significantly with C/N ratio increased, suggesting that the supply of organic carbon is insufficient for the reproduction of denitrifiers. At C/N ratios above 8, negligible increase in the abundances of the three genes was noted, which indicated that the denitrifier population size may be limited by the electron acceptor (${NO}_{3}^{-}$-N). Besides, excessive organic carbon may be beneficial for other kinds of heterotrophic microorganisms (Kraft et al., 2014), inhibiting the growth of denitrifiers. Therefore, a C/N ratio of 8 was recommended.

During the incubation period, the absolute abundance of *nirK* was always less than that of *nirS*, which may be ascribed to the insufficient organic carbon or unsuitable NaAc for the growth of denitrifiers containing *nirK*. As the functions of *nirS* and *nirK* are similar (Stouthamer, 1992), denitrifiers containing *nirS* predominated in nitrate removal. The abundance of *nosZ* positively increased with the C/N ratio (**Table 2**), suggesting that organic carbon is important for denitrifiers containing *nosZ*. Inwood et al. (2007) confirmed that the increase in organic carbon reduced the production of N_2_O. In the present study, an obvious increasing trend of *nirS*, *nirK*, and *nosZ* abundances (**Figure 3** and **Table 2**) provided direct evidence of an ecological association and symbiotic relationship among the denitrifier communities at the molecular level (functional genes).

### Effect of C/N Ratio on the Diversity and Composition of Denitrifiers

To investigate the effect of C/N ratio on the diversity and composition of denitrifiers, the Shannon index, Simpson index, and sequence were examined. The results showed that the C/N ratio was not significantly correlated with the diversity index (including Shannon and Simpson indices) with respect to *nirS* (*r* = -0.26, 0.038; *P* = 0.672, 0.956, respectively), but a slight correlation with the diversity index with respect to *nirK* (*r* = 0.75, -0.80; *P* = 0.11, 0.10, respectively). This could possibly be owing to the higher C/N ratio being unfavorable for the growth of denitrifiers containing *nirS*, but favorable for the growth of denitrifiers containing *nirK*. Chen et al. (2010) noted that denitrifiers containing *nirK* were more sensitive to the C/N ratio than denitrifiers containing *nirS*. Furthermore, Yoshida et al. (2009) found that *nirS*-containing denitrifiers in rice field were not sensitive to C/N ratio. Besides, the diversity of denitrifiers containing *nirK* may be unimportant for the whole system owing to their low absolute abundance. Therefore, we presumed that the diversity of denitrifiers was not important for the apparent nitrate removal, but it is important for the stability of ecosystem.

**FIGURE 4:**
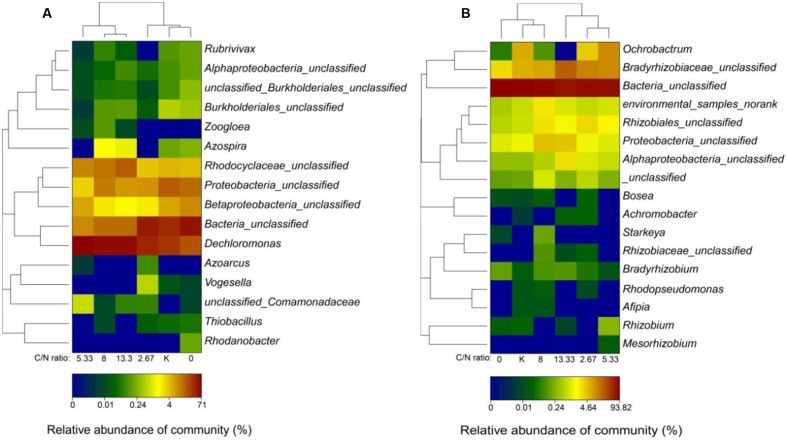
Hierarchical cluster analysis of the communities of **(A)** *nirS*-type and **(B)** *nirK*-type denitrifiers. The *y*-axis indicates clustering of 300 most abundant OTUs (3% distance) in the reads. The OTUs were arranged based on genus. Sample communities were clustered based on the complete linkage method. The color intensity of the scale indicates the relative abundance of each OTU read. Relative abundance was defined as the number of sequences affiliated with that OTU divided by the total number of sequences per sample. *K* represents control samples (incubated with distilled water).

The community composition, relative abundance, and molecular phylogenetic relationship of denitrifiers containing *nirS* were determined by hierarchical clustering of *nirS* (**Figure 4A**). The results revealed that Proteobacteria (46.00–90.28%) and β-Proteobacteria were dominant at the phylum and class level, respectively, similar to those observed in a previous study on groundwater (Chu and Wang, 2013). Eight known genera (i.e., *Dechloromonas*, *Azoarcus*, *Azospira*, *Rubrivivax*, *Thiobacillus*, *Vogesella*, and *Zoogloea*) were detected, among which *Dechloromonas* was predominant. At C/N ratios below 5.33, bacteria-unclassified (35.72–48.33%) and *Dechloromonas* (18.81–38.89%) were predominant, while at C/N ratios above 5.33, *Dechloromonas* (56.18–70.60%) was dominant. With further increases in the C/N ratio, the percentage of Rhodocyclaceae-unclassified increased. It has been reported that the majority of *Dechloromonas* could reduce nitrate by utilizing ferrous iron or organic carbon as the electron donor and without nitrite accumulation (Coates and Achenbach, 2004). Moreover, studies on denitrifier community in activated sludge indicated that Rhodocyclaceae could easily utilize organic carbon for efficient denitrification (Khan et al., 2002; Ginige et al., 2005). In addition, the presence of *Rubrivivax*, which lacks nitrate reductase and needs external nitrite for denitrification, could mitigate the accumulation of nitrite (Saarenheimo et al., 2015).

By using hierarchical clustering of *nirK*, the community compositions, relative abundance, and molecular phylogenetic relationship of denitrifiers containing *nirK* were explored (**Figure 4B**). At the phylum level, bacteria-unclassified (67.96–93.82%) was dominant, similar to that reported in a previous research on soil (Bremer et al., 2007). At the genus level, nine known genera (namely, *Achromobacter*, *Afipia*, *Bosea*, *Bradyrhizobium*, *Mesorhizobium*, *Ochrobactrum*, *Rhizobium*, *Rhodopseudomonas*, and *Starkeya*) were detected. The proportion of genera affiliated with Bradyrhizobiaceae-unclassified was obviously higher in the system with a C/N ratio of 13.33 (21.45%) than that in the other groups (3.19–12.50%), which was owing to sufficient electron donor (Guo et al., 2011; Wang Z. et al., 2014). At C/N ratios below 5.33, the percentage of *Ochrobactrum* increased with increasing C/N ratio (*r* = 0.988, *P* = 0.099); however, at C/N ratios above 5.33, the percentage of *Ochrobactrum* decreased with the increase in C/N ratio (*r* = -0.762, *P* = 0.448). Summarily, Proteobacteria and *Dechloromonas* were predominant at the phylum and genus level, respectively, and *Dechloromonas* may play a dominant role in nitrate removal.

## Conclusion

This study investigated the influence of C/N ratio on nitrate removal from the sediments of freshwater ponds. The nitrate removal efficiency increased with increases in the C/N ratio. When the C/N ratio was higher than 8, adequate denitrification without ${NO}_{2}^{-}$-N accumulation was achieved. When the C/N ratio was less than 8, excessive nitrite accumulation occurred. Hence, a C/N ratio of 8 is recommended for nitrate removal from pond sediments. When the C/N ratio was increased from 0 to 13.3, the absolute abundance of *nirS*, *nirK*, and *nosZ* genes correspondingly increased. The positive correction between nitrate removal rate and nitrogen functional genes further confirmed that different nitrogen transformation processes were coupled to synergistically contribute to nitrogen removal at the molecular level (functional genes). *Dechloromonas* dominated the overall process of nitrate removal in systems with a C/N ratio above 5.33, while the majority of *nirK*-type denitrifiers were unclassified.

## Author Contributions

RC designed and conducted the experiment, accomplished the first draft, and corrected the article. MD and JH provided some valuable suggestion for this article. XH proposed the topic and corrected the manuscript.

## Conflict of Interest Statement

The authors declare that the research was conducted in the absence of any commercial or financial relationships that could be construed as a potential conflict of interest.
