# Supplementary material for: Enhancing Nitrate Removal from Freshwater Pond by Regulating Carbon/Nitrogen Ratio
Source: Front Microbiol. 2017 Sep 8;8:1712. doi: 10.3389/fmicb.2017.01712 (PMC5596099; doi:10.3389/fmicb.2017.01712)
Supplement: Supplementary file 1 [file Presentation_1.PDF]

Supplementary Material for the Manuscrip "Enhancing Nitrate Removal from Freshwater Pond by Regulating Carbon/Nitrogen Ratio"

Rong Chen[1,2], Min Deng[1], Xugang He[1,3,4*], Jie Hou[1*]

1 *College of Fisheries, Huazhong Agricultural University, Wuhan 430070, P. R. China.*

2 *School of Environmental Studies, China University of Geosciences, Wuhan, 430074, P. R. China.*

3 *Freshwater Aquaculture Collaborative Innovation Center of Hubei Province, Wuhan, 430070, P. R. China.*

4 *Hubei Provincial Engineering Laboratory for Pond Aquaculture, Wuhan, 430070, P. R. China.*

---

* Corresponding author (xgh@mail.hzau.edu.cn)

Material and method

Table S1 Primers of target genes used in PCR analysis

Target gene	Primer	Primer sequence (5’-3’)	Amplification size (bp)	Reference
nirS	nirScd3aF	GTSAACGTSAAGGARACSGG	425	(Throbäck et al., 2004)
	nirSR3cd	GASTTCGGRTGSGTCTTGA		
nirK	nirKFlaCu	ATCATGGTSCTGCCGCG	473	(Hallin and Lindgren, 1999)
	nirKR3Cu	GCCTCGATCAGRTTGTGGTT		
nosZ	nosZ1527F	CGCTGTTCHTCGACAGYCA	250	(Scala and Kerkhof, 1998)
	nosZ1773R	ATRTCGATCARCTGBTCGTT		

Supplementary References for Table S1

Hallin, S., Lindgren, P. E. (1999). PCR detection of genes encoding nitrite reductase in denitrifying bacteria, *Appl. Environ. Microbiol.* 65, 1652-1657.

Scala, D. J., Kerkhof, L. J. (1998). Nitrous oxide reductase (*nosZ*) gene-specific PCR primers for detection of denitrifiers and three *nosZ* genes from marine sediments, *FEMS Microbiol. Lett.* 162, 61-68. doi: 10.1111/j.1574-6968.1998.tb12979.x

Throbäck, I. N., Enwall, K., Jarvis, Å., Hallin, S. (2004). Reassessing PCR primers targeting *nirS*, *nirK* and *nosZ* genes for community surveys of denitrifying bacteria with DGGE. *FEMS Microbiol. Ecol.* 49, 401-417. doi: 10.1016/j.femsec.2004.04.011

Table S2 Protocols and parameters of target genes used in qPCR analysis

Target gene	Primer (μL)	Programs
nirS	0.4	pre-denaturation at 95 ℃ for 30 s, denaturation at 95 ℃ for 5 s, annealing at 57 ℃ for 30 s, extension at 72 ℃ for 40 s, and terminal extension at 72 ℃ for 7 min
nirK	0.5	pre-denaturation at 95 ℃ for 30 s, denaturation at 95 ℃ for 5 s, annealing at 55 ℃ for 40 s, extension at 72 ℃ for 50 s, and terminal extension at 72 ℃ for 7 min
nosZ	0.4	pre-denaturation at 95 ℃ for 30 s, denaturation at 95 ℃ for 5 s, annealing at 58 ℃ for 50 s, extension at 72 ℃ for 30 s, and terminal extension at 72 ℃ for 7 min

Results and discussion

Table S3 The correlation of nitrate removal efficiency with C/N ratio

Culture time (d)	Correlation coefficient	Sig.
5	0.92	0.00
10	0.91	0.00
15	0.78	0.00
20	0.78	0.00
25	0.70	0.00
30	0.68	0.01

Table S4 The correlation of nitrite accumulation with C/N ratio

Culture time (d)	Correlation coefficient	Sig.
5	-0.85	0.00
10	-0.92	0.00
15	-0.84	0.00
20	-0.87	0.00
25	-0.82	0.00
30	-0.82	0.00

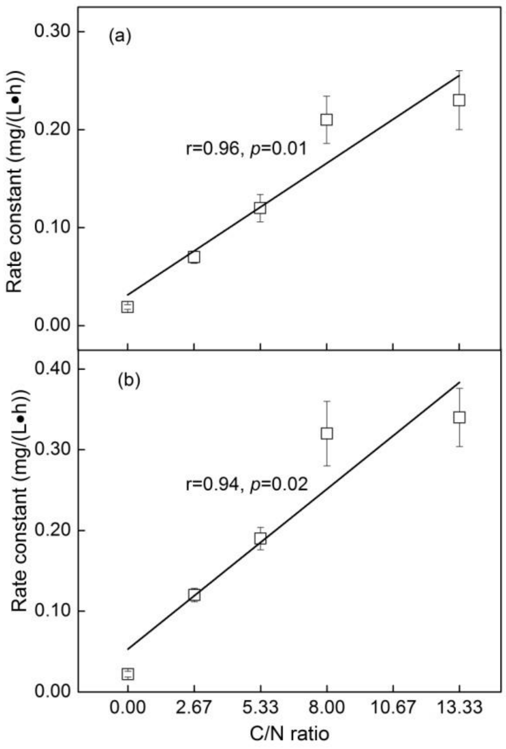

Fig. S1 Correlation between C/N ratio and rate constant of nitrate removal at 15th day (a) and 30th day (b).
